# Recent advances and future challenges in gene therapy for hearing loss

**DOI:** 10.1098/rsos.230644

**Published:** 2023-06-14

**Authors:** Ana E. Amariutei, Jing-Yi Jeng, Saaid Safieddine, Walter Marcotti

**Affiliations:** ^1^ School of Biomedical Science, University of Sheffield, Sheffield S10 2TN, UK; ^2^ Neuroscience Institute, University of Sheffield, Sheffield S10 2TN, UK; ^3^ Institut Pasteur, Université Paris Cité, Inserm, Institut de l'Audition, F-75012 Paris, France

**Keywords:** hearing, hearing loss, deafness, cochlea, hair cell, gene therapy

## Abstract

Hearing loss is the most common sensory deficit experienced by humans and represents one of the largest chronic health conditions worldwide. It is expected that around 10% of the world's population will be affected by disabling hearing impairment by 2050. Hereditary hearing loss accounts for most of the known forms of congenital deafness, and over 25% of adult-onset or progressive hearing loss. Despite the identification of well over 130 genes associated with deafness, there is currently no curative treatment for inherited deafness. Recently, several pre-clinical studies in mice that exhibit key features of human deafness have shown promising hearing recovery through gene therapy involving the replacement of the defective gene with a functional one. Although the potential application of this therapeutic approach to humans is closer than ever, substantial further challenges need to be overcome, including testing the safety and longevity of the treatment, identifying critical therapeutic time windows and improving the efficiency of the treatment. Herein, we provide an overview of the recent advances in gene therapy and highlight the current hurdles that the scientific community need to overcome to ensure a safe and secure implementation of this therapeutic approach in clinical trials.

## 1. Introduction

The sense of hearing is key for our daily life, ranging from the appreciation of music to the ability to localize sound in space and being able to communicate with friends and family. Severe or profound deafness in children impacts on the development of spoken language and the ability to read, which has consequences for academic performance and ultimately employability. In cases where hearing loss occurs later in life, such as with progressive hearing loss, people tend to become socially isolated, leading to feelings of loneliness and depression. When untreated, midlife-acquired hearing loss represents the largest modifiable risk factor for cognitive decline and dementia [1,2].

Currently, hearing loss is the most common sensory disorder in humans and represents one of the most prevalent chronic dysfunctions in older adults. Based on the World Health Organization [3], about 432 million adults and 34 million children worldwide have disabling hearing impairment, which is predicted to reach 700 million by 2050 (about 10% of the world's population). With age, the percentage of people with hearing impairment rises to about 60% for adults over the age of 50 [4]. Although the aetiology underlying hearing loss can be quite diverse, genetic mutations cause about 70–75% of congenital deafness ([5,6]; Hereditary Hearing Loss homepage: https://hereditaryhearingloss.org/). Much less is known about the genetics underlying progressive, late-onset hearing loss, since it is influenced by environmental factors such as noise, drugs and past infection [7–9]. However, studies of twins and families have shown that genetic predisposition represents an important risk factor for age-related hearing loss (ARHL), reporting heritability of up to 0.9 [10–12]. Moreover, genome-wide association studies in adults affected by hearing loss are continuously discovering new candidate genes for ARHL. These studies are also identifying new variants in several genes known to be involved in childhood deafness [13–16].

Although hearing loss can target different cell types within the auditory pathway (e.g. [17–19]), one of the most common forms of hearing loss is caused by the damage and/or loss of the sensory hair cells and the associated auditory neurons that make synaptic connections within the cochlea, which is collectively called sensorineural hearing loss (SNHL: [8,20–22]). Currently, the only available options for ameliorating SNHL are hearing aids or cochlear implants. While these are very beneficial (e.g. [23–25]), they cannot restore important features of natural sound perception, such as temporal processing and frequency tuning, especially in noisy environments, leading to poor speech recognition. Recent developments in the methods available for modifying and correcting genetic abnormalities, together with our increased understanding of the genes responsible for deafness, have meant that gene-based therapy has become an appealing approach for addressing hearing loss. Although gene therapy is the primary focus of this article, other alternative therapeutic approaches are being developed for hearing loss, such as the use of stem cells and other molecular strategies [26–28].

## 2. Gene-based therapy for hereditary hearing loss

Gene therapy is primarily aimed at either replacing, suppressing or editing faulty genes to treat disease [29,30]. The cochlea is well suited for these therapeutic approaches due to its anatomical isolation and the presence of the blood-labyrinth barrier, which separates it from the systemic blood circulation. This unique feature makes it an ideal target for gene therapy as it helps to reduce the risk of any potential off-target systemic dissemination. In addition, the therapeutic vector can be delivered directly into the fluid space of the cochlea where it can easily diffuse throughout its entire length to reach the targeted cell type. Among the different gene-based approaches, the replacement of a faulty gene with a normal copy has been the most widely used to treat monogenic hearing loss [31,32]. Currently, more than 20 pre-clinical studies using knockout mice or mouse models for human forms of deafness have shown varying degrees of functional hearing recovery following the replacement of the defective gene in the cochlea (up-to-date list available in: [32]). The success of some of these initial studies in tackling recessive deafness DFNB9 [33,34], a form of congenital deafness caused by mutation in the *Otof* gene, has facilitated the development of the first adeno-associated virus (AAV) vector for *OTOF*-mediated deafness in human patients. In late 2022, three separate clinical trials have been approved for DFNB9: OTOF-GT by Sensorion (https://www.sensorion.com/en/our-approach/restore-treat-prevent/), DB-OTO by Decibel Therapeutics (https://www.decibeltx.com/pipeline/) and AK-OTOF by Akouos (https://akouos.com/our-focus/).

Despite the recent success, further research is necessary before AAV-based gene therapy can be FDA and EMA approved and used as a therapeutic treatment for hearing loss. This is primarily dictated by the limitations and challenges associated with this recently developed approach in the field. Most of the research reporting successful hearing recovery in mice suffering from hereditary deafness has focused on genes primarily contributing to the development and/or function of the postnatal mouse cochlea, e.g. *Tmc1* [35,36], *Vglut3* [37,38], *Otof* [33,34] and *Pjvk* [39]. However, the reinstatement of hearing function has proven to be more difficult for AAV-based replacement of genes required for early stages of cochlear development. For example, hearing recovery is absent or very minimal for genes involved in the initial growth of the hair cell stereociliary bundles, such as *Eps8* or those causing Usher syndrome when mutated (*Harmonin*: Usher 1C; *Sans*: Usher 1G; *Whrn*: Usher 2, *Clarin-1*: Usher 3A: [40–45]). This could be due to the low transduction rate of the AAVs in the hair cells at more mature stages or possibly because the cochlea has incurred irreversible damage making gene therapy treatment ineffective. The earlier delivery of genes *in utero,* instead of neonatal stages, appears to improve the transduction rate of the inner hair cells and to some extent functional hearing recovery (e.g. *Gjb6*: [46,47]). In addition, several lines of evidence show that the transduction efficiency of the AAV gene replacement approach in the adult mouse cochlea is reduced compared to that performed at early postnatal stages [45,48]. Therefore, it may only be possible to achieve a comparable restoration in the mature cochlea by using AAV variants that possess a high tropism to hair cells.

Another important consideration is the different developmental timelines between mice and humans. In humans, the auditory system is fully functional at birth, with auditory startle reflexes appearing from around 24 weeks gestation [49]. Therefore, the majority of cochlear maturation in humans occurs *in utero*. In mice, the cochlea is immature at birth and requires at least another two–three weeks to reach full maturity [50]. The consequence of this is that, even though the use of mice has provided strong evidence for the viability of gene therapy to treat at least some forms of deafness, which are important findings, it is still difficult to predict the likely outcome in humans. A related complication is that almost all the pre-clinical work has been performed in pre-hearing mice, which, if translated to the equivalent therapeutic window in humans, would fall at around 20 weeks gestation. Of course, any therapeutic intervention during human pregnancy would pose several potential risk factors and safety issues for both baby and mother. This issue could present a substantial hurdle for FDA and EMA approval and for the recruitment of patients to clinical trials.

An additional solution would be to identify the forms of congenital deafness that would be amenable to gene therapy at later stages, once the cochlea has fully developed (curing hearing loss rather than preventing it). This could also include the identification of deafness genes that, when defective, cause a more progressive deterioration of cochlear function, such as those involved in ARHL. In both cases, the gene therapy approach would benefit from a longer therapeutic window for intervention. The success of this approach will depend on further fundamental and pre-clinical research being carried out, including the identification of novel AAV variants that can efficiently target inner ear hair cells, as well as the optimization of their delivery into both neonatal and adult mice. Moreover, further development is required in the use of cell-specific promoters, which will also help to reduce the ectopic expression of genes in off-target cell types. Finally, more research is required to determine the possible side effects of the therapy in the cochlea in terms of inflammation and immunotoxicity. The immune response to AAVs in humans is still largely unknown [51], but ongoing clinical trials in the eye indicate that viral gene therapy may trigger an adaptive immune response [52–54].

## 3. Conclusion

Considering that pre-clinical research for the use of gene-based therapy to treat hearing loss only began about 15 years ago, there has been substantial progress made with three groups advancing towards clinical trials for the *OTOF* gene. Given current knowledge and progress, AAV gene therapy represents a promising prospect for ameliorating, preventing or even curing hereditary hearing loss that affects millions of people worldwide. However, we have to remain aware that several challenges need to be overcome before any gene therapy for hearing loss can be applied to patients, which could take several years after the successful completion of clinical trials (e.g. AAV-based gene therapy for the eye was FDA-approved 10 years after successful clinical trials: [55]). Some of these challenges include overcoming limitations imposed by critical therapeutic time windows, the use of more specific capsids and promoters to increase transduction efficiency and specificity of treatment, finding the most effective delivery methods, and ways of expanding the modest packaging capacity of viral vectors. In addition, we need a better understanding of the potential side effects of the treatment, including the immune response to it and the longevity of the treatment, for which very little is known, especially with regard to use in humans. The current research and planned clinical trials will be instrumental for addressing at least some of these unknowns.

## Data Availability

This article has no additional data.
